# A Novel Deep Learning and Ensemble Learning Mechanism for Delta-Type COVID-19 Detection

**DOI:** 10.3389/fpubh.2022.875971

**Published:** 2022-07-08

**Authors:** Habib Ullah Khan, Sulaiman Khan, Shah Nazir

**Affiliations:** ^1^Department of Accounting and Information Systems, College of Business and Economics, Doha, Qatar; ^2^Department of Computer Science, University of Swabi, Swabi, Pakistan

**Keywords:** hybrid deep learning, Delta-type COVID-19, VGG16, ensemble learning technique, AI

## Abstract

Recently, the novel coronavirus disease 2019 (COVID-19) has posed many challenges to the research community by presenting grievous severe acute respiratory syndrome coronavirus 2 (SARS-CoV-2) that results in a huge number of mortalities and high morbidities worldwide. Furthermore, the symptoms-based variations in virus type add new challenges for the research and practitioners to combat. COVID-19-infected patients comprise trenchant radiographic visual features, including dry cough, fever, dyspnea, fatigue, etc. Chest X-ray is considered a simple and non-invasive clinical adjutant that performs a key role in the identification of these ocular responses related to COVID-19 infection. Nevertheless, the defined availability of proficient radiologists to understand the X-ray images and the elusive aspects of disease radiographic replies to remnant the biggest bottlenecks in manual diagnosis. To address these issues, the proposed research study presents a hybrid deep learning model for the accurate diagnosing of Delta-type COVID-19 infection using X-ray images. This hybrid model comprises visual geometry group 16 (VGG16) and a support vector machine (SVM), where the VGG16 is accustomed to the identification process, while the SVM is used for the severity-based analysis of the infected people. An overall accuracy rate of 97.37% is recorded for the assumed model. Other performance metrics such as the area under the curve (AUC), precision, F-score, misclassification rate, and confusion matrix are used for validation and analysis purposes. Finally, the applicability of the presumed model is assimilated with other relevant techniques. The high identification rates shine the applicability of the formulated hybrid model in the targeted research domain.

## Introduction

Globally, the COVID-19 pandemic continues to have catastrophic effects on human lives. According to the WHO reports, about 4.8 million people died due to this outbreak and more than 19 million people were infected by this pandemic (1). To combat this outbreak, researchers around the world presented novel-based models for the identification of this outbreak's symptoms. But, the regional-based varying symptoms and emergence of a new type of virus (COVID-19 type) pose daunting challenges for the researchers to counter. The recrudescence of the new Delta COVID-19 virus swirled the researchers and practitioners due to its high resistance against human mutants and high death rates in a short period. After analyzing the integrating capabilities and achieving satisfactory results laterally for numerous research problems, the researchers and practitioners suggested artificial intelligence (AI)- and machine learning (ML)-based models for the detection of COVID-19. Keeping in view the reality that AI techniques and models have left no stone unturned, Togaçar et al. (2) suggested a deep learning model for the identification of COVID-19 with the help of Social Mimic Optimization and structured chest X-ray images based on fuzzy color and stacking techniques. Alazab et al. (3) suggested a deep learning technique for the recognition of COVID-19 infection.

Ismail and Sengür assumed deep learning models for the prediction of COVID-19 detection (4). Chang et al. (5) presented deep learning for diagnosing COVID-19 infection using chest X-ray images. Karhan and Akal presented a convolutional neural network (CNN)-based architecture for the identification of COVID-19 using X-ray images (6). Their model consists of a residual network (ResNet) for classification and recognition purposes. A recognition rate of 78% is recorded for their model, which reflects high misclassification rates. This high misclassification shows that this model classifies 22% of normal persons as COVID-19-infected patients. Khan et al. (7) presented a three-dimensional (3D) deep learning model for diagnosing COVID-19 using CT images. Their model consists of 3D-ResNets, 3D-DenseNets, C3D, I3D, and a long-term recurrent convolutional network (LRCN). They performed their analysis of two different datasets (CC-19 and COVID-CT). Keeping in view the shortage of PCR kits and colossal demands, Anwar and Zakir proposed reverse transcriptase-PCR (RT-PCR) for identifying the COVID-19 infection using deep learning (8). For evaluating the performance of their model, they used three different strategies with varying learning rates: (1) limiting the learning rate when the model performance stops increasing (reduce on the plateau), (2) constant learning rate, and (3) cyclic learning rate. A diagnosing rate of 89.7% is recorded for their model.

After evaluating the extant, it was realized that almost every automatic diagnosing model is affected by low recognition rates, high simulation time, complexities, or requires more data to train (data hunger architectures), and many others. Keeping in view these issues in mind, a hybrid model is presented in this study for accurately addressing these problems and performing timely diagnosing and identification tasks. The technical contribution of the proposed research study includes:

- **Optimum diagnosing model**—the development of an optimum recognition model for accurate identification and diagnosing of Delta-type COVID-19-infected personnel. This hybrid model consists of a visual geometry group 16 (VGG16) and a support vector machine (SVM). The VGG16 model achieves the classification of the infected chest images, while the SVM classifier commits the statistical operations to calculate the severity of an individual patient. This statistical analysis will help assign a bed (for treatment purposes) to a patient on a priority basis that has a higher probability of being recovered.
- **Performance analysis**—the validity of the proposed model with other state of the art techniques to evaluate the applicability and integrity of the formulated hybrid model in the selected research domain. Also, different performance metrics such as F-score, accuracy, misclassification rate, specificity, the area under the curve (AUC), and confusion matrix are used for performance analysis purposes.
- **Variant-based validation**—after analyzing the extant, some models show poor performance for small datasets, while some models show poor capabilities for symptoms-based variations (different virus types). Keeping in view these challenges, the proposed hybrid model concluded with an outstanding performance for the COVID-19 variant (Delta type). Based on these performance capabilities, it can be applied to other variants of the severe acute respiratory syndrome coronavirus 2 (SARS-CoV-2) infections. Furthermore, this is non-data-driven architecture and even outperformed a small number of X-ray samples.
- **Patient's health status evaluation**—to the best of the research team's knowledge, much study is reported on the X-ray-based COVID-19 detection and diagnosing purposes (9–11), but there is no study reported to check the severity of a particular patient using statistical and analytical techniques. This severity evaluation of a particular patient will ultimately help the state agencies and practitioners with different aspects:
  ° To calculate the strengths of this outbreak in a specific region based on patients' historical- and severity-based information.
  ° Also, this will help the doctors in performing priority-based treatment of specific patients. As we know, when an outbreak hits, mainly the senior personnel or the ones already facing some diseases (heart issues, diabetics, cough, or other lung problems) are more susceptible to being infected and may face severe conditions. With low hardware resource requirements, a timely decision model is required that helps the practitioners and doctors to identify the severe cases automatically.
  ° This premature analysis and modeling will help the state agencies to carry out their routine activities without interrupting their economy, hospital structure, and other daily activities.

The rest of the article is coordinated as follows. Section Literature Review outlined the background and research study reported in the extant. It also outlined the gaps in the extant and explained how the proposed model will address all these challenges in the extant. Section Methodology outlined the proposed experimental setup followed for diagnosing the COVID-19-infected personnel. In Section Results and Discussion of the article, the results and discussion section are briefly explained that outlined the performance analysis of the suggested model. Also, this section briefly explained the applicability of the proposed research study by comparing its results with other avant diagnosing models presented in the extant. Section Conclusion of the article outlined the conclusion of this research study pursued by the recommendation and future research study in Section Implications.

## Literature Review

At the end of 2019, the first case of a novel infection, latterly codenamed as COVID-19, was reported in Wuhan city in China. This virus expanded worldwide and affected millions of people in a short span of time (12–14). The aftershock of this outbreak results in the emergence of new variants (Delta type, Omicron, etc.) in different regions. The Delta-type variation of COVID-19 recently emerged in India and was disseminated quickly around India and even its symptoms were sensed in the neighboring countries, including Sri Lanka, Maldives, and southern regions of Pakistan. This variant concluded with high mortalities even in short span of time. The researchers and practitioners were busy synthesizing the symptoms and their underlined catastrophic effects on human lives that a new variant of COVID-19 emerged in South Africa's Gauteng province, where the achingly mutated breed of the virus was first figured out and was researched that it is more potent than other variants in escaping anterior immunity. This variant is codenamed as Omicron (15). Maria Van Kerkhove, the WHOs technical team lead on COVID-19, stated in a video posted on Twitter “*This variant has a large number of mutations and some of these mutations have some worrying characteristics*.”

These symptoms-based variations and consequently high mortalities puzzle the research community and practitioners to counter this outbreak. A smart machine learning model is required capable of not only countering Delta-type COVID-19, but can combat the afterward variants with optimum capabilities. Inspired by the applicability of AI and machine learning techniques in diverse domains such as traffic flow predictions (16) and smart object detection in smart homes for ensuring high security (17), many researchers proposed deep learning for classifying and recognizing the COVID-19 infection. Multiple convolutional neural network (CNN) classification models such as ResNet50V2, DenseNet201, and Inceptionv3 are suggested for identifying patients with COVID-19 using chest X-ray images (3, 18, 19). Experimental results for their models were calculated using only 468 images. It shows poor performance for larger datasets and its simulation time becomes very high for comparatively larger databases. Qiao et al. (20) proposed the focal loss-based neural network ensemble mechanism for COVID-19 identification purposes in this research study. They performed the simulations on X-ray images. They presented a novel-based approach, but their model shows small performance metrics values (low precision values of 0.783360.07, recall values of 0.860960.03, and F1-score values of 0.81686 0.03).

Salman et al. (21) suggested an artificial intelligence-based deep learning model for diagnosing the COVID-19 virus by applying chest images. They performed their analysis on 130 different images. Their model performed an outstanding performance for this small amount of data, but fails in classifying comparatively larger datasets. The research article presented by Loey et al. (22) shows a generative adversarial network (GAN) with deep transfer learning for COVID-19 recognition using chest X-ray images. They performed their experimental study using 307 images for four different types of classes. Their model achieved 80.6% in much smaller testing accuracy, especially in healthcare applications. Additionally, the number of images used for experimental purposes is much smaller than a standard testing and validation model. Similarly, Chang et al. (5) have proposed VGG16 for diagnosing COVID-19 infection using chest X-ray images. An overall accuracy rate of 78% is achieved for this model, which is comparatively much smaller and ultimately it reflects a high misclassification rate. Anwar and Zakir proposed RT-PCR for identifying COVID-19 infection using deep learning models (8). For evaluating the performance of their model, they used three different strategies with varying learning rates. This model helps in reducing the dependency on PCR testing kits and colossal demands. An overall accuracy rate of 89.7% is recorded for their model, which ponders the incapability of this model in the proposed research domain. Dhiman and Kaur suggested a high optimization model for industrial engineering problems after inspiration by bioinspired-based techniques. Inspired by the collaborative behavior of spotted hyenas, Dhiman and Kumar suggested a novel metaheuristic algorithm named as Spotted Hyena Optimizer (SHO). The applicability of the SHO-based model is validated using different 29 state of the art benchmark functions (23, 24). Based on their performance analysis, they concluded that their models outperform the available models in the extant. Kaur et al. (25) presented the concept of metaheuristic paradigm for global optimization using a novel Tunicate Swarm and NOVA tests.

After studying the literature and assessing their capabilities and limitations, it was concluded that most of the techniques developed in the literature are either tested on small datasets or affected by low accuracy rates, while some of these models are good for COVID-19, but fail in diagnosing the variants of the SARS-CoV-2-like COVID-19 (Delta type, Omicron, etc.). These difficulties in variant-based diagnosing are because of diverging symptoms and their underlined effects on human bodies. A smart machine learning model is required to combat all these challenges and perform optimum diagnosing for different variant-base infections. Keeping in view these questions in mind, a hybrid model is formulated in this study and was concluded with an outstanding performance for the COVID-19 variant (Delta type). Based on the performance capabilities, it can be concluded that this model can be applied to other variants of the SARS-CoV-2 infections. Furthermore, this is low data-hunger architecture and even outperformed a small number of X-ray samples.

## Methodology

**Figure 1:**
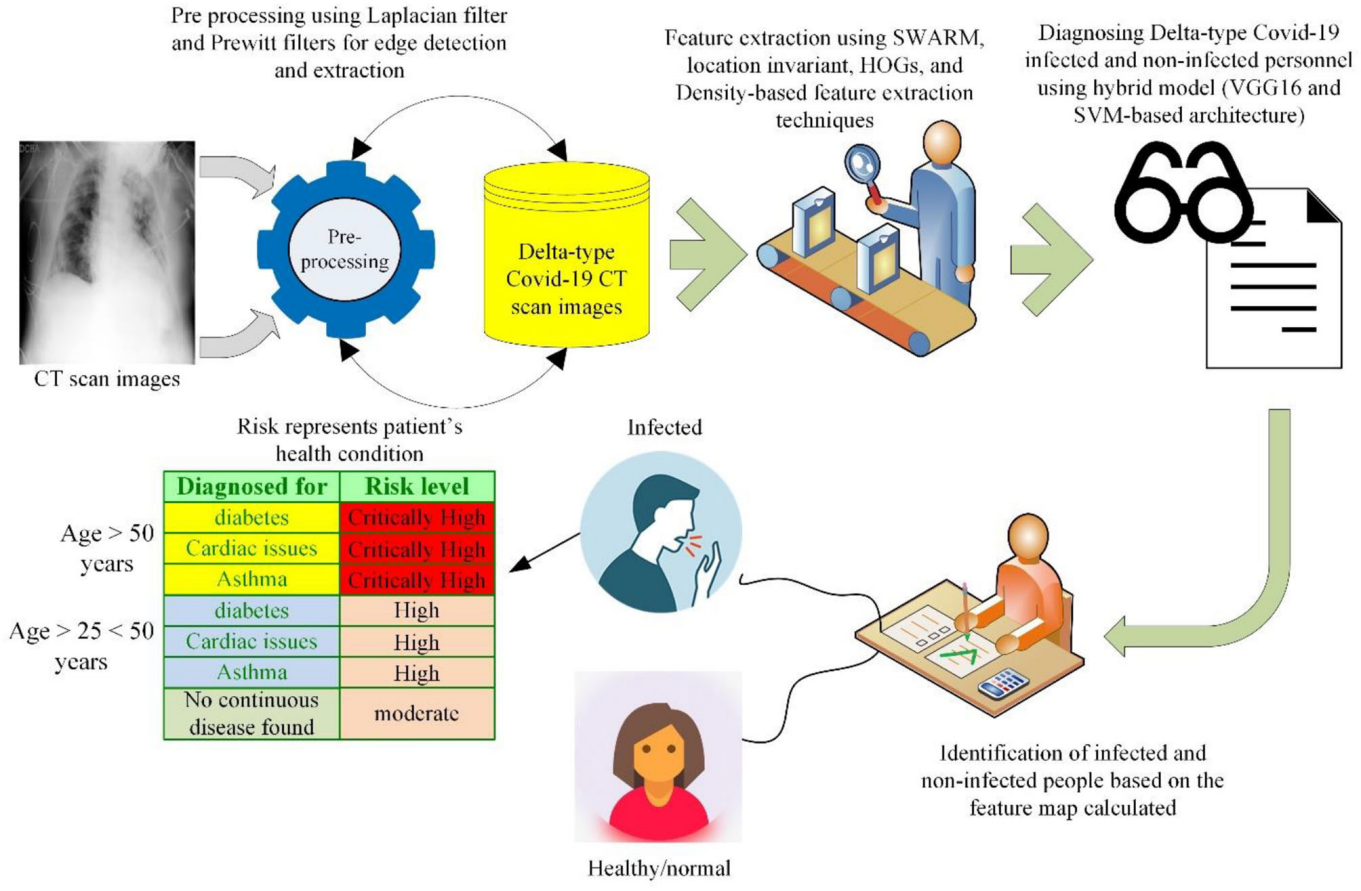
Experimental setup of the proposed study.

Figure 1 represents the experimental setup followed for the proposed research study. In this research study, a hybrid deep learning model is suggested for identifying Delta-type COVID-19-infected patients. This hybrid model comprises VGG16 and SVM classifiers, where the VGG16 architectures perform the classification tasks, while the SVM performs the statistical analysis to calculate the severity (health status) of patients. In this research model, different preprocessing and feature extraction techniques are used for ensuring high identification rates. To clarify the X-ray images and laterally assure high recognition rates, this research study has proposed Prewitt and Laplacian filters to extract the edge information of the infected region. Then, feature extraction techniques such as the Swarm-based feature extraction technique (26), histogram of oriented gradients (HoG), and location invariant features are proposed to calculate the feature map. In the X-ray images, most of the time, the resultant images are blurred, scaled, rotated, and many others and most of the feature extraction techniques fail in accumulating optimum astute value in such cases, but invariant feature extraction techniques are prominent in these cases because their feature accumulation capabilities are never affected by rotation and scaling (27).

After applying these feature extraction techniques, a fused feature map (features calculated by HoG, location invariant technique, and Swarm-based feature extraction technique) is developed that is provided to the hybrid model formulated in this research study. The diagnosing and classification task is performed using this feature map.

**Figure 2:**
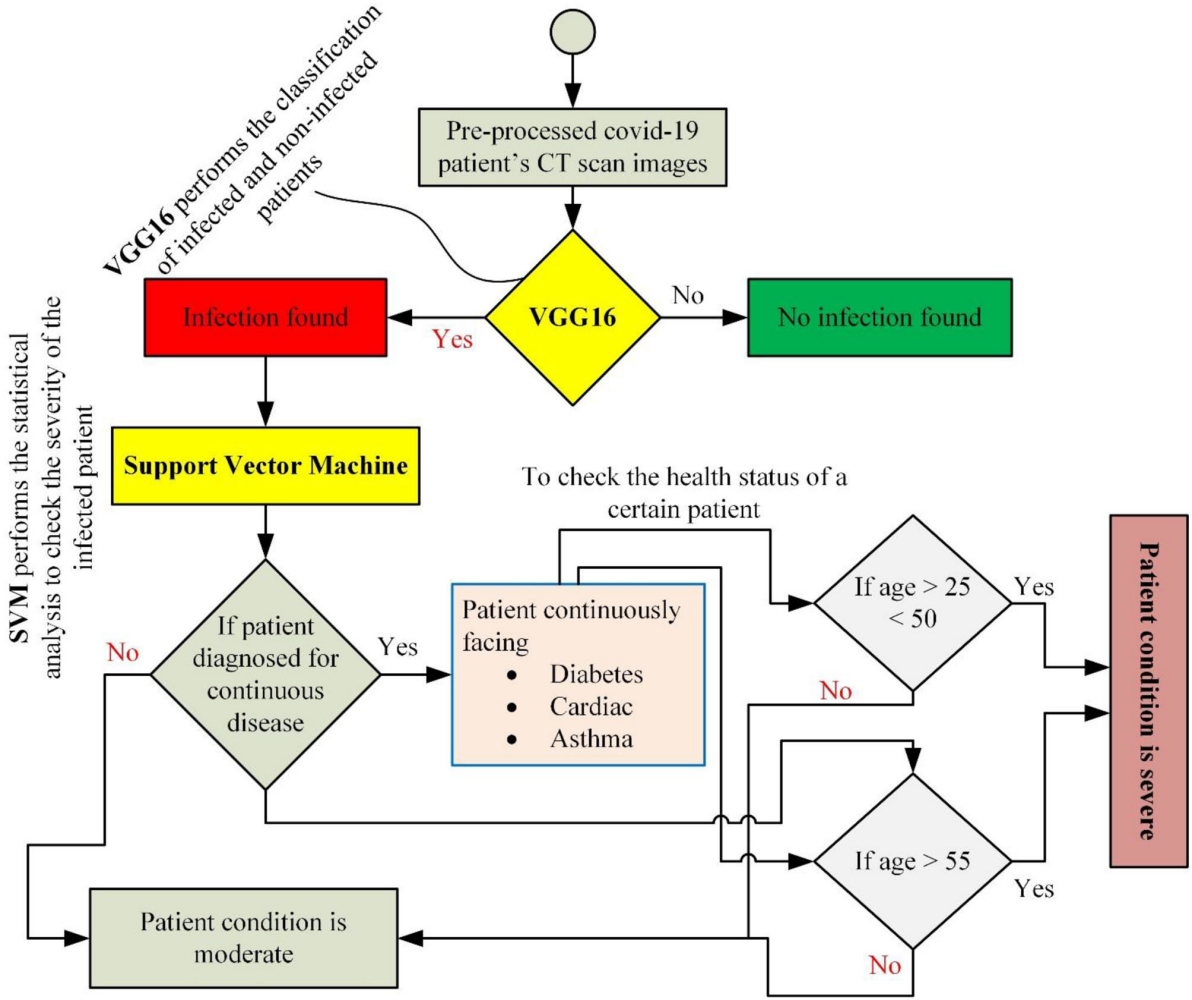
A stepwise solution was followed to implement the proposed hybrid diagnosing model.

After executing the classification task on the feature map, if a person is diagnosed with the infection, then his/her severity is measured using statistical analysis based on different parameters such as age, suffering from a continuous disease (diabetes, cardiac problems, asthma, etc.), and health status. Being binary in nature (28), the SVM outperforms the identification of critically severe and severe conditions. The overall statistical operations followed for identifying the health status (severity) of a certain patient are given in a flowchart diagram in Figure 2. This stepwise solution is followed for identifying the severity of each patient based on the classification results generated by the VGG16 classifier.

## Results and Discussion

**Figure 3:**
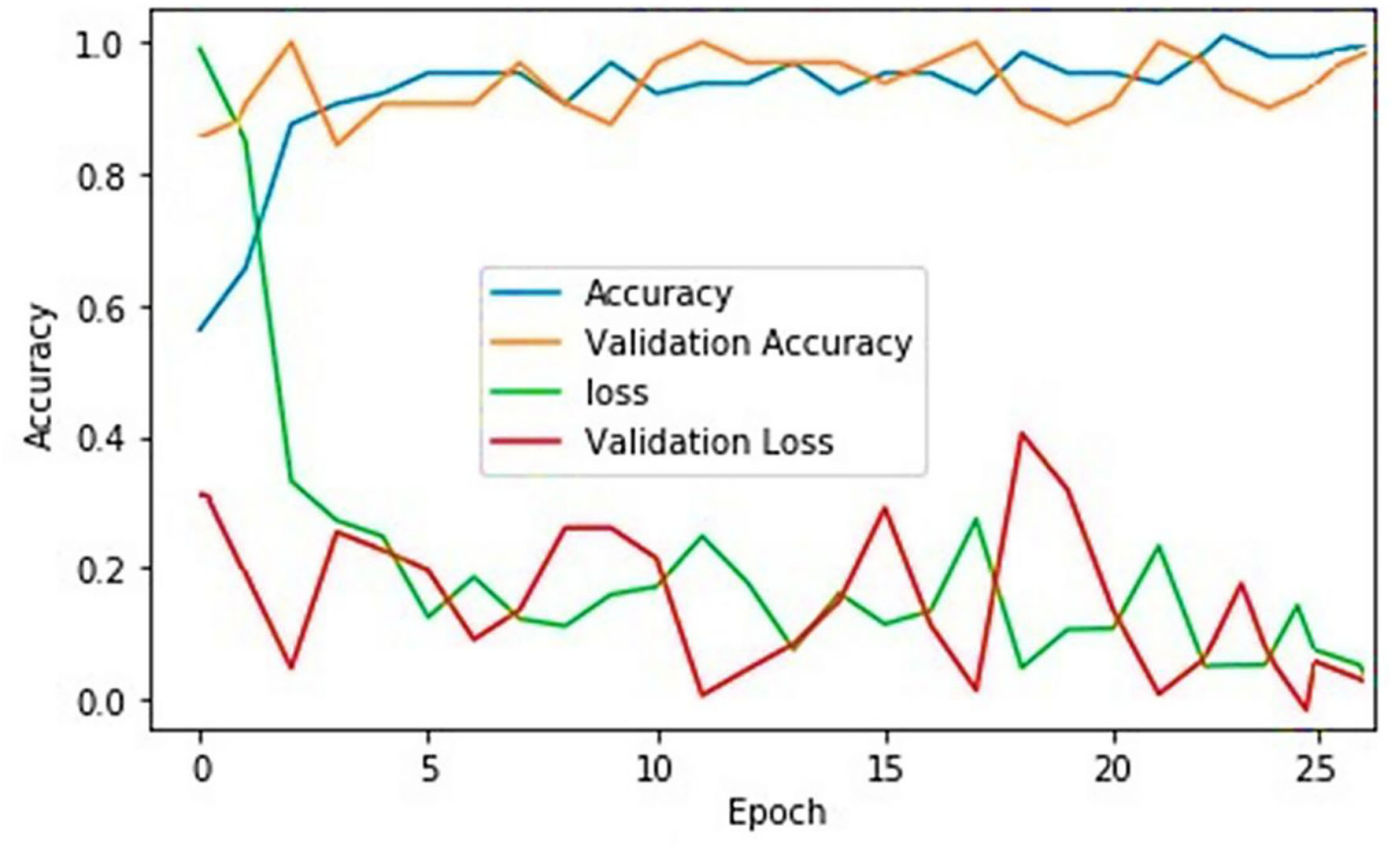
Performance results of the suggested hybrid deep learning technique.

This part of the article shows the experimental results generated by the proposed hybrid model after applying the manipulated feature map. This study outperformed by simulating an overall accuracy rate of 97.37%, as shown in Figure 3. For this research study, a dataset^1^ is selected from the GitHub library freely available for research and simulation purposes and some other Delta-type infection images are collected in the group from multiple patients in the hospital (Lady Reading Hospital, Peshawar, Pakistan) and the WHO reports (15). The details about the number of samples used for the experimental study are given in Table 1. A total of 5,844 samples were used for this experimental study. The details regarding the number of samples, gender, age, and infection details are given in Table 1. The accumulated samples in Table 1 represent the number of samples collected from the hospital. This eminent recognition rate reflects the applicability of the proposed model for the identification of Delta-type COVID-19. Even based on these high-performance values, it can be concluded that this hybrid model can be employed for the identification of other COVID-19 variants.

**Table 1:** Details about the images database used for the experimental study.

**Age-wise distribution**	**Infection-based distribution**			**Gender-wise distribution**		**Total number of samples selected from**	
	**Cardiac**	**Diabetes**	**Asthma**	**Male**	**Female**	**GitHub library**	**Accumulated samples**
Age ≥ 50	1,020	924	1,644	59.8%	40.2%	2,600	988
Age>25 <50	411	433	1,374	52.1%	47.%	1,700	518
Age>1 ≤ 25	5	0	33	63.7%	36.3%	35	3

From Table 1, it can be easily observed that this disease has a comparatively low infection in youngers (age > 1 ≤ 25 years). Different parameters are selected for the validation and applicability analysis of the proposed research study. The information regarding these parameters is shown in Table 2.

**Table 2:** Set of parameters defined for the proposed hybrid model.

**S. No**	**Parameter**	**Value**
1.	Number of inputs	100 × 100 × 1, 200
2.	Filter	64
3.	Total number of layers	5
4.	Number of hidden layers	3
5.	Kernel function size	(3 × 3)
6.	Batch size	32
7.	Epoch size	50
8.	Optimizer	Adam
9.	Activation function	Rectified Linear Unit and Tanh

The statistical assessment was performed using the formulated research model (SVM) for describing the severity of any particular patient, as shown in Table 1 (since the statistical analysis is performed only, if a patient is diagnosed with infection). There is no low level (risk level) defined for the patient because someone's life and health status are more important than anything. This severity-based analysis and underlined risk level are calculated after consulting a professional caretaker. The caretaker provided detailed information on the infection attack on aged and continuously diseased patients. Table 3 outlines these metrics with a full description.

**Table 3:** Severity-based analysis.

**Age (years)**	**Type of continuous disease diagnosed**			**Health status (risk level)**	**Output and comments**
	**Cardiac issues**	**Diabetes**	**Asthma**		
Age ≥ 50	**✓**	**✓**	**✓**	Critically high	Patient requires proper medication
Age ≥50	***×***	**✓**	**✓**	Critically high	and isolation. Also, he/she needs a
Age ≥ 50	**✓**	***×***	**✓**	Critically high	significant attention of the caretakers.
Age ≥ 50	**✓**	**✓**	***×***	Critically high	
Age ≥ 50	**✓**	***×***	***×***	Critically high	
Age ≥ 50	***×***	**✓**	***×***	Critically high	
Age ≥ 50	***×***	***×***	**✓**	Critically high	
Age>25 <50	**✓**	**✓**	**✓**	High	Patient requires proper medication
Age>25 <50	***×***	**✓**	**✓**	High	and isolation. Also, he/she needs a
Age>25 <50	**✓**	***×***	**✓**	High	significant attention of the caretakers.
Age>25 <50	**✓**	**✓**	***×***	High	
Age>25 <50	**✓**	***×***	***×***	High	
Age>25 <50	***×***	**✓**	***×***	High	
Age>25 <50	***×***	***×***	**✓**	High	
Age>1≤25	***×***	***×***	**✓**	High	Patient requires medication and attention of the caretaker.
Age>1≤25	***×***	***×***	***×***	Moderate	Patient needs only proper medication.

*Colors are used to differentiate in-between different age categories*.

**Figure 4:**
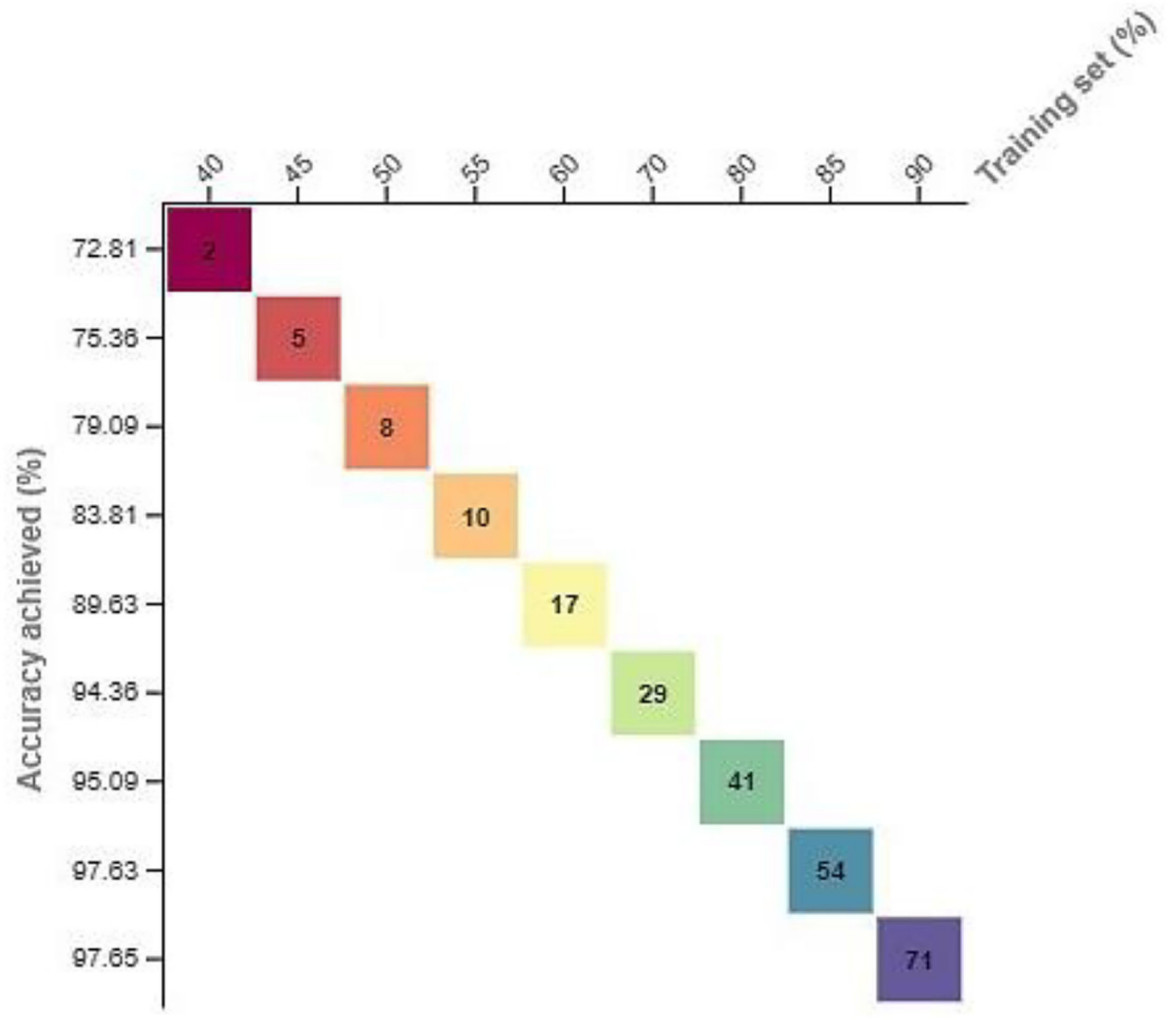
Evolution of the suggested model using different training and test sets.

**Figure 5:**
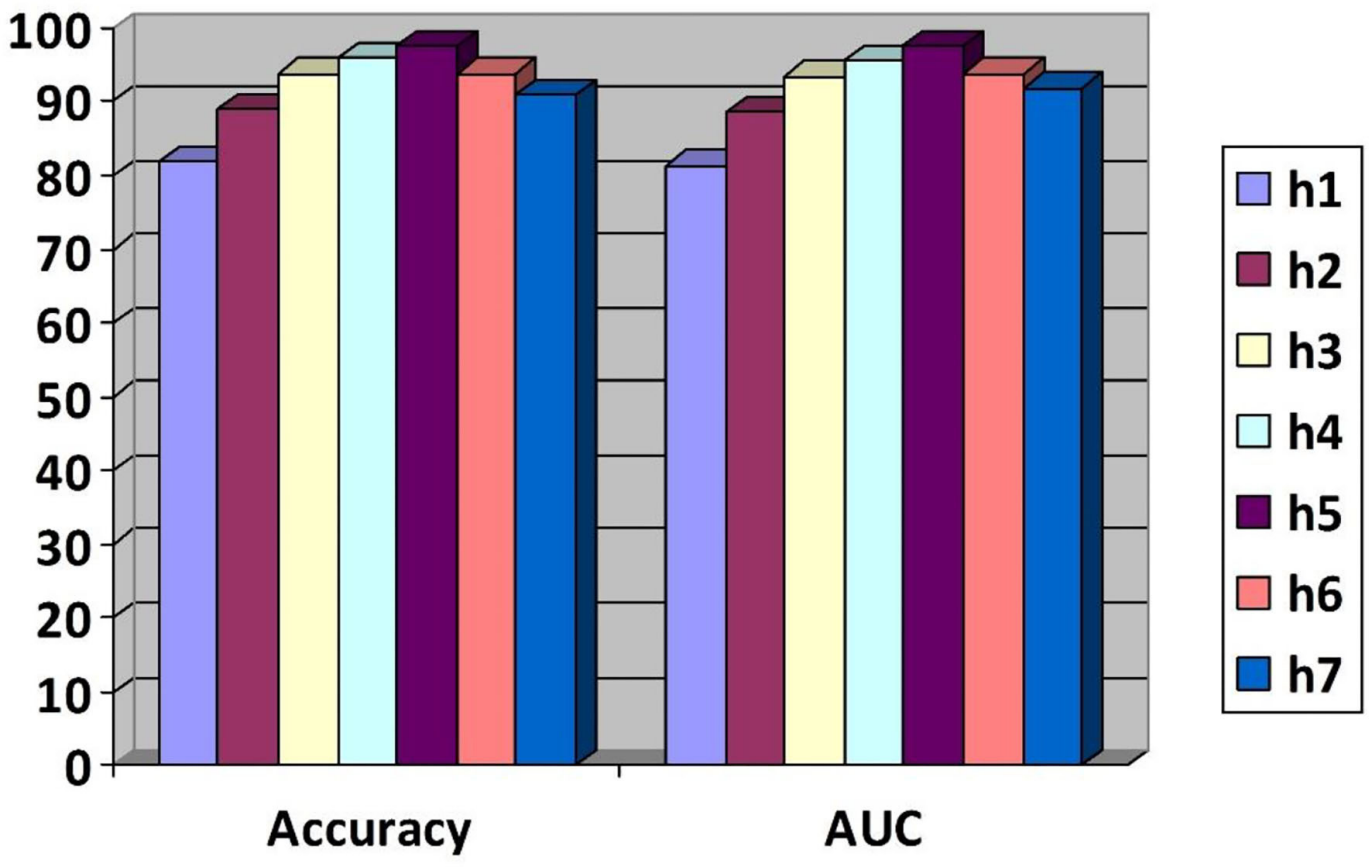
The area under the curve (AUC) and accuracy values are based on a varying number of hidden layers.

The formulated research model is also validated using different training and test sets and the different number of hidden layers (h). The simulated results are shown in Figures 4, 5. The squares represent the accuracy values based on the training set, while the numbers in the square boxes represent the simulation time in minute(s). The simulation results are simulated with HP Core i3 3rd generation laptop with Intel processor [that is why the simulation time is recorded more comparative to the graphics processing unit (GPU)-based processors].

To perform these experiments, a training set of a varying number of samples is used. These samples started by dividing the overall data into 40% training data and the remaining test data and then 45% training data and the remaining test data and so on. At the same time, the accuracy of the corresponding technique is noted to validate the applicability of the proposed model. The corresponding results are shown in Figure 4. Using this varying number of samples, the performance of the proposed model is also evaluated using the different numbers of hidden layers based on the AUC values and accuracy as a performance indicator. Based on the simulated results, it was concluded that this model outperformed by generating the high AUC and accuracy values.

From Figures 4, 5, it is observed that by increasing the number of hidden layers or the training set, the corresponding time consumption and accuracy of the model increase. But, after hidden layer 5, the accuracy of the model starts decreasing, which reflects the complexity of the circuit and high simulation cost.

**Figure 6:**
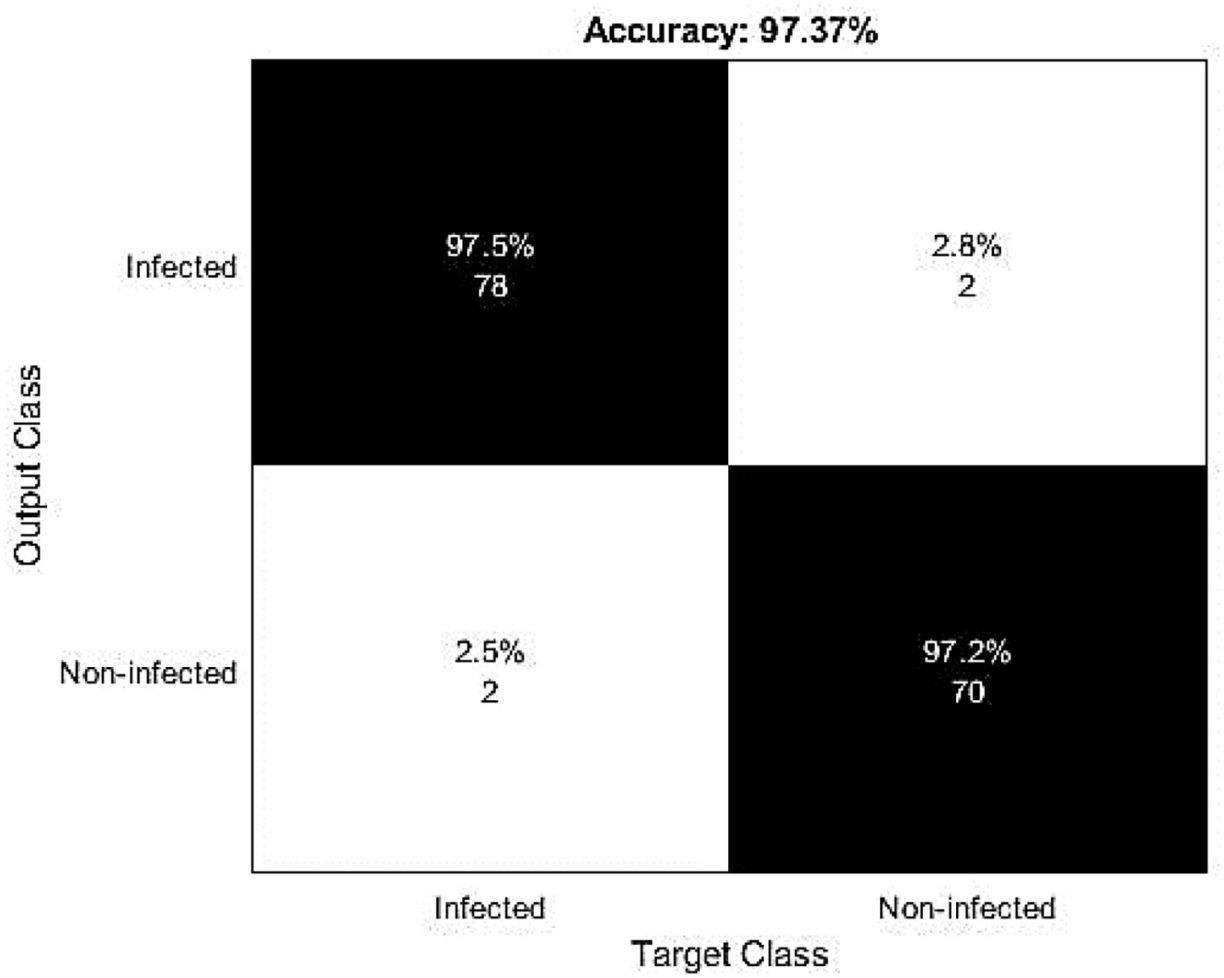
The confusion matrix reflects the recognition capabilities of the proposed research study.

**Figure 7:**
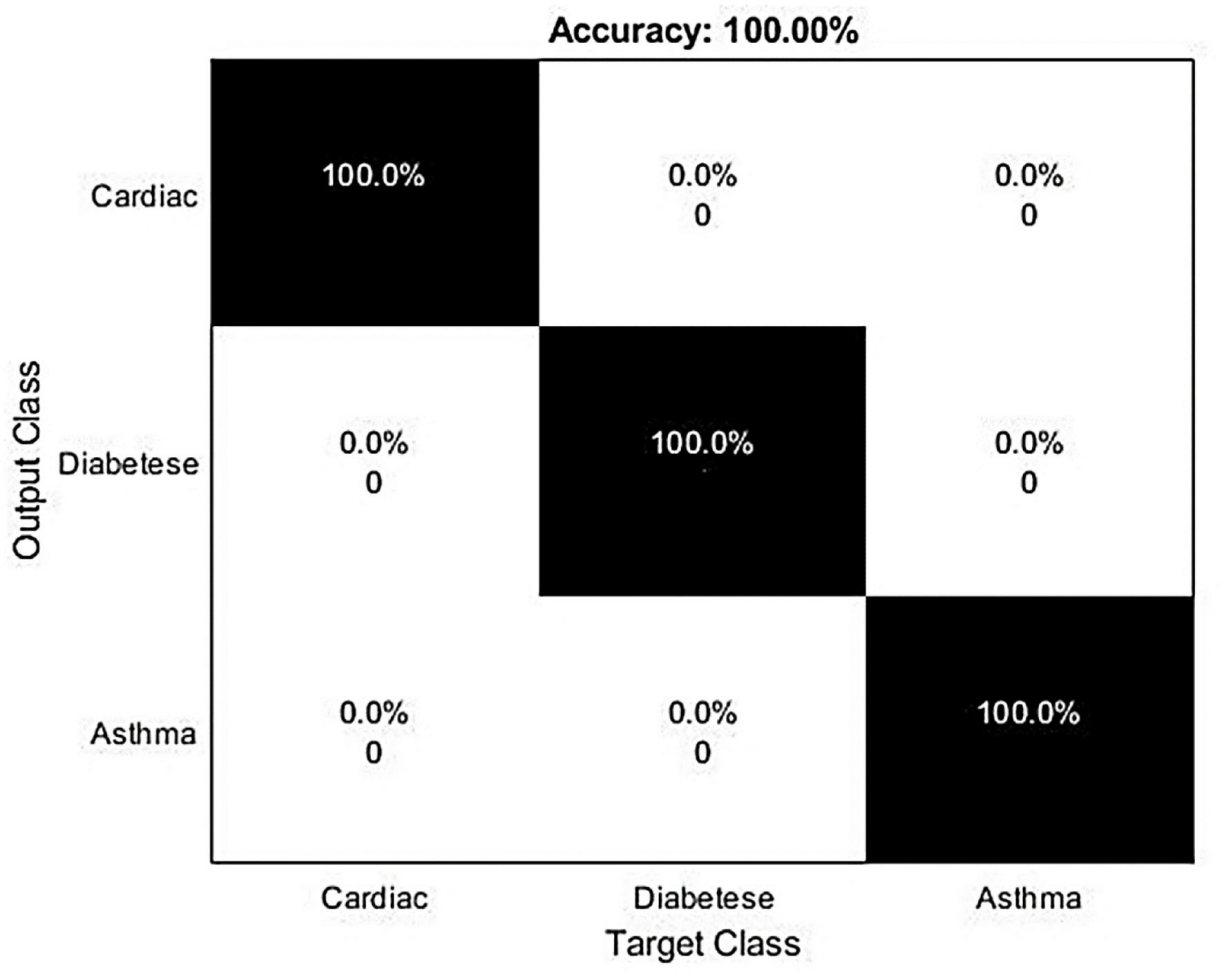
Confusion matrix for severity-based analysis of the infected patients.

The confusion matrix of this research study is given in Figure 6. The small number of false positive and false negative represents the materiality of the proposed hybrid deep learning model for the identification of Delta-type COVID-19 infection. Figure 7 contains the diagnosing details of Delta-type COVID-19 infection using VGG16.

A total of 5,844 images were used (1,948 images for every three different classes with both the infected and non-infected states). The corresponding results based on infection and non-infection are given in Figure 6. These results were evaluated based on the clinical historical information and the results were found correct, as shown in Figure 6.

For this experimental and simulation study, we used an HP Core i3 machine. So, to avoid high-time consumption, a small number of samples are used for training and testing purposes. Some CT scan images are highly blurred and it is more difficult for the proposed model to accurately recognize that ultimately causing misclassification. While plotting a confusion matrix to evaluate the capabilities of this model for the severity-based analysis, a 100% accuracy is recorded for the SVM-based severity analysis. This high accuracy rate reflects the applicability of the formulated research model in this research domain and it ultimately shows that if a patient has cardiac issues, asthma, or diabetes, then the patient has a high risk of mortality and needs proper treatment and high attention of the caretaker.

**Figure 8:**
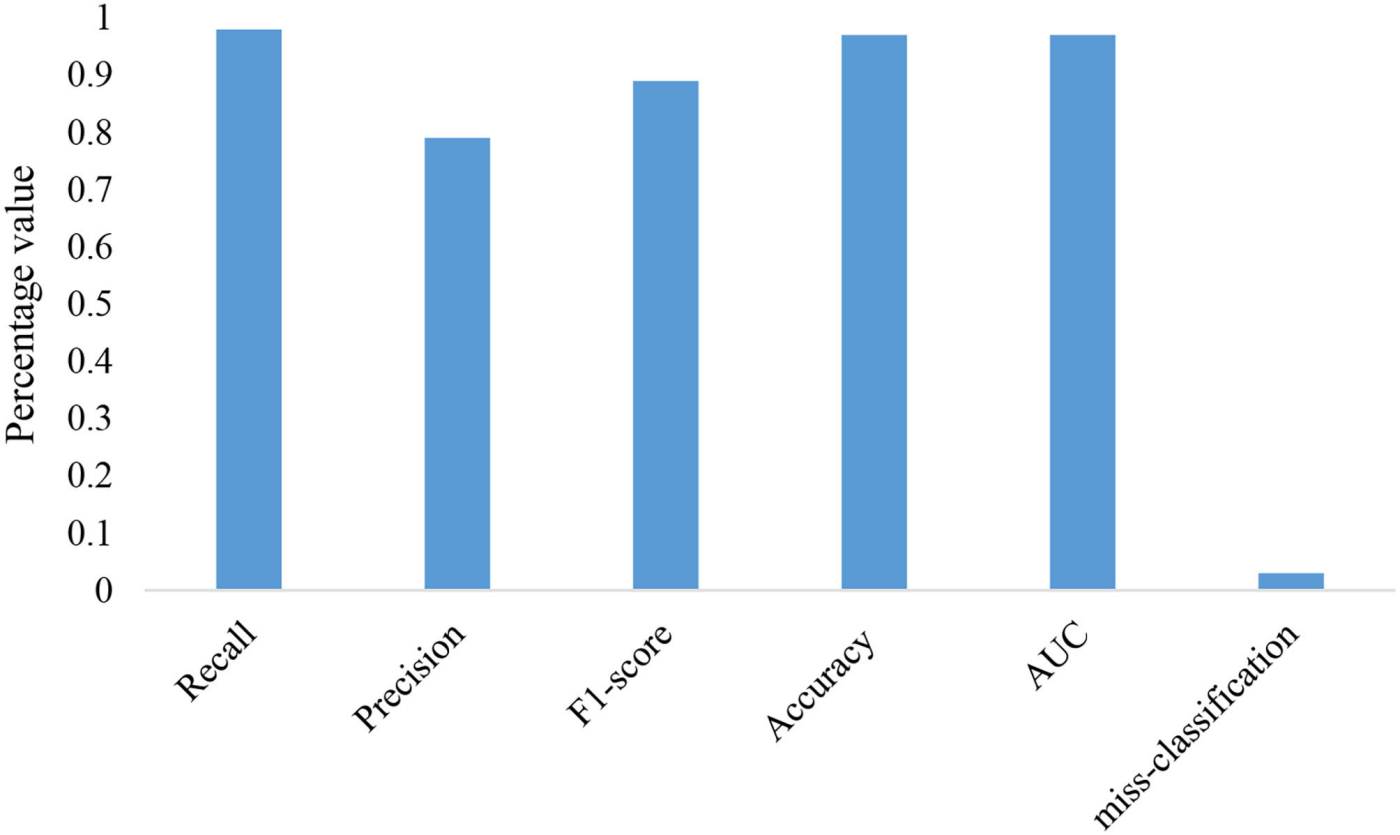
Evolution of the proposed research model.

Other performance metrics such as F-score, misclassification rate, precision, recall, and other are used for evaluation purposes and the corresponding results are given in Figure 8. The small misclassification rate reflects the applicability of our model.

For performance validation purposes, different state of the art models are suggested for testing the applicability of the formulated research study in the targeted research domain. A list of these models is given below:

- **ResNet**—residual network or in short ResNet is the modified version of the CNN architecture extensively used for image classification and recognition purposes in diverse domains, including hyperspectral image classification (29) and digital image-based steganalysis (30). Keeping in view these applications of the ResNet, the recognition capabilities of the targeted research study are tested with the ResNet classifier.
- **CNN 1D Net**—this classification tool is extensively used in the extant for calculating the temporal dependencies and has proven its capabilities in the image classification and recognition domains. The CNN 1D Net is also suggested for validating the performance of the targeted model.
- **DenseNet**—Densely Connected Convolutional Network or Dense Convolutional Network or simply DenseNet is the modified version of the CNN architecture. Recently, it was observed that CNN architectures can be primarily deeper, higher accuracy, and more efficient to train if they comprise shorter connections between the layers closer to the input and the output layers (31). Keeping in view these recognition capabilities, the researchers proposed DenseNet in diverse domains such as computer vision and machine learning applications.

**Figure 9:**
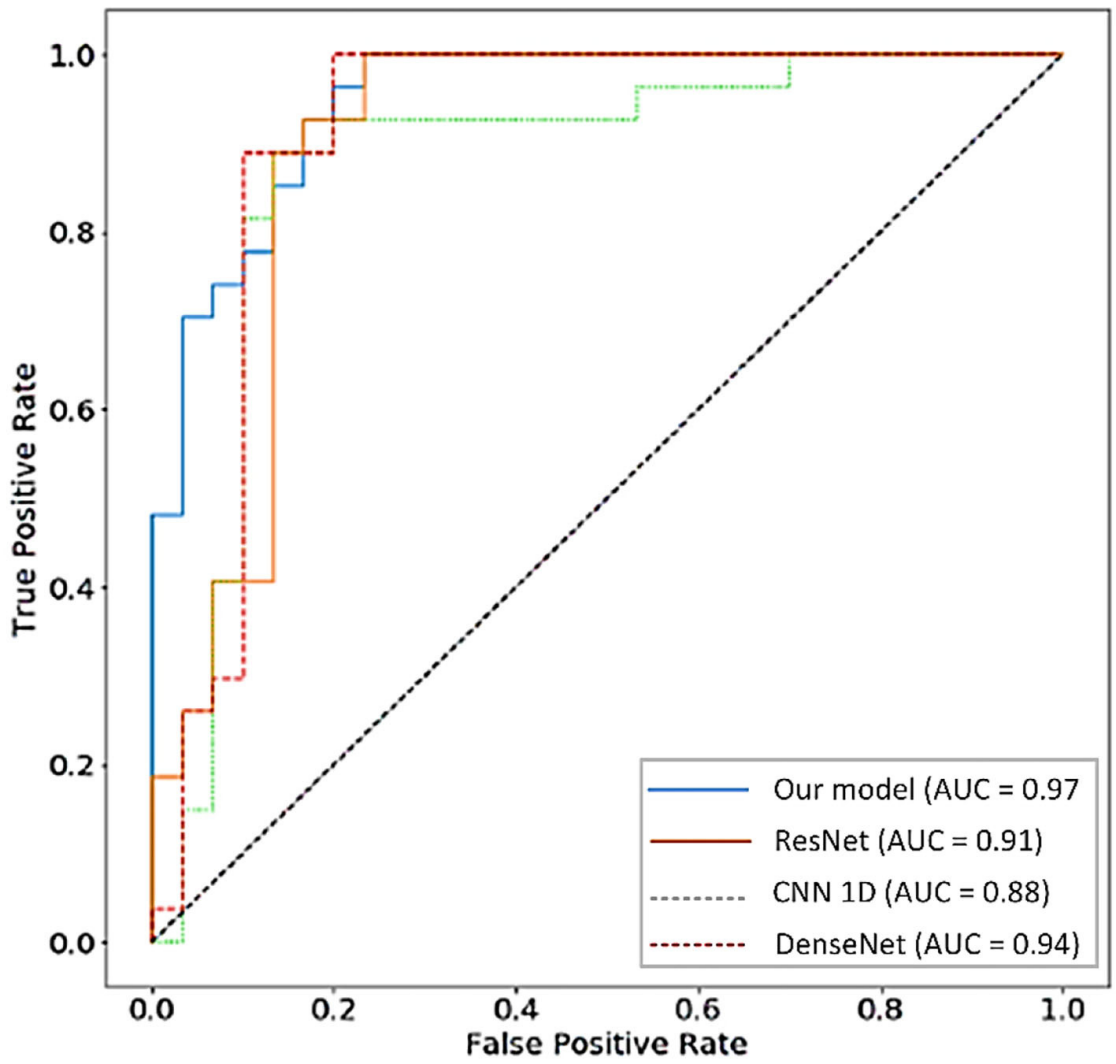
The AUC values-based performance analysis of this research model with convolutional neural network one-dimensional (CNN 1D Net), residual network (ResNet), and Dense Convolutional Network (DenseNet) models.

The applicability of the proposed model is compared with these state of the art models using the area under the curve (AUC) values as a performance metric. The higher AUC value for the proposed model shows the usability of the proposed model in the COVID-19 variants diagnosing and identification. The corresponding results are given in Figure 9.

Performance capabilities of the formulated research model are also validated using other evaluation and assessment metrics such as F-score, misclassification rate, and accuracy. The corresponding results are given in Table 4. From Table 3, it is concluded that after evaluating all the models on different performance metrics, our model outperformed by generating the high AUC, accuracy, and other performance values that muse the integrity of the selected research study in the targeted research topic.

**Table 4:** Performance comparison with the targeted classification models.

**Techniques**	**Recall**	**Precision**	**F1-score**	**Accuracy (%)**	**AUC**
ResNet	0.93	0.86	0.90	0.92	0.91
DenseNet	0.93	0.86	0.87	0.95	0.94
CNN 1D Net	0.76	0.82	0.85	0.86	0.88
Proposed model	0.98	0.97	0.94	0.97	0.97

## Conclusion

At the end of 2019, the emergence of SARS-CoV-2 and its aftermath of different variants such as Delta-type COVID-19 and Omicron puzzled the research community with its distinct symptoms and high mortality capabilities. Researchers around the world affiliated with both the government and nongovernment organizations contributed by developing different diagnosing and identification models for the inspection of COVID-19 and its variants, but most of these models have limitations in the form of time consumption, high implementations costs, capabilities to a specific type of infections, data hunger (performs well if a huge amount of data is available otherwise fails), and many others. In order to tackle this issue, a hybrid deep learning model is produced in this research for the automatic inspection of Delta-type COVID-19. A recognition rate of 97.37% is generated for the automatic identification of Delta-type infection based on the X-ray images. In this hybrid deep learning model, VGG16 and SVM are used for identification and classification purposes. The VGG16 is used for the classification and identification of the infected patients based on the X-ray images, while the SVM is used for the severity-based analysis of the infected patient based on the statistical analysis. These statistical analyses include age, suffering from continuous diseases (diabetes, cardiac issues, and asthma), and physical status.

The applicability of the presented model is tested with other state of architectures suggested by researchers in the proposed domain. These models include CNN 1D Net, DenseNet, and ResNet. The proposed model outperformed using the F-score, the AUC values, accuracy, precision, and misclassification rate as evaluation metrics. The high accuracy, the AUC, F-score, precision, and small misclassification rates compared to the selected state of the art models muse the pertinence of the formulated model in the targeted research problem. To check the applicability of this model for other variations, two different datasets of COVID-19 (CC-19 and COVID-CT) were selected to test its applicability other than Delta-type infection. The proposed hybrid model proved its applicability by generating high identification rates and it ultimately reflects the adaptability of this model to various types of infection.

## Implications

This research study has unlimited applications, especially in evaluating the severity of a certain infected patient. This severity-based analysis will help the caretakers and practitioners to provide healthcare facilities to the severe patient on an emergency basis to restrict high mortalities. The proposed model can be easily implemented and installed in hospitals, medical stores, and other healthcare centers to perform automatic diagnosing based on X-ray images.

## Data Availability Statement

The original contributions presented in the study are included in the article/supplementary material, further inquiries can be directed to the corresponding author.

## Author Contributions

HK: concept, data collection, and project supervision. SK: methodology and writing. SN: discussion and analysis. All authors contributed to the article and approved the submitted version.

## Funding

This research work is supported by Qatar National Library and Qatar University Internal Grant No. IRCC-2021-010. The results obtained herein are solely the obligation of the writers.

## Conflict of Interest

The authors declare that the research was conducted in the absence of any commercial or financial relationships that could be construed as a potential conflict of interest.

## Publisher's Note

All claims expressed in this article are solely those of the authors and do not necessarily represent those of their affiliated organizations, or those of the publisher, the editors and the reviewers. Any product that may be evaluated in this article, or claim that may be made by its manufacturer, is not guaranteed or endorsed by the publisher.

## Footnotes

^1^ https://github.com/owid/covid-19-data/tree/master/public/data/
